# Psychometric Validation of the Persian Version of the Experiential Avoidance Rating Scale (EARS) and Its Application in Assessing Psychological Inflexibility in Persian-Speaking Populations

**DOI:** 10.1186/s40359-025-03364-x

**Published:** 2025-10-02

**Authors:** Maedeh Imany, Shahram Mohammadkhani, Haniyeh Sadat Atashipour, Parastoo PourHosseini

**Affiliations:** Kharazmi University, Tehran, Iran

**Keywords:** Experiential avoidance, Measurement, Psychological flexibility

## Abstract

This study evaluated the psychometric properties of the Persian version of the Experiential Avoidance Rating Scale (EARS) to provide a reliable measure of experiential avoidance for Persian-speaking populations. A cross-sectional design was employed with 700 university students from Tehran (M = 27.71 years, SD = 9.35; 77.9% female). The EARS was translated via a forward–backward method to ensure cultural and conceptual equivalence. Confirmatory factor analysis supported a single-factor model with an excellent fit (χ^2^(9) = 41.81, CFI = 0.98, RMSEA = 0.072). The scale showed good internal consistency (α = 0.81). Concurrent validity was supported by significant correlations with the Acceptance and Action Questionnaire-II (r = 0.60) and the Brief Experiential Avoidance Questionnaire (r = 0.40). A weaker correlation was found with the Multidimensional Psychological Flexibility Inventory’s Experiential Avoidance subscale (r = 0.14), which may reflect differences in construct focus and format. Convergent validity was evidenced by correlations with PTSD symptoms and negative affect, whereas divergent validity was confirmed by a nonsignificant relationship with traditional masculinity norms. One item showed a notably low factor loading (β = 0.26). This finding may reflect cultural nuances in item interpretation. Despite this, the item was retained to preserve consistency with the original EARS. These findings indicate that the Persian EARS is a reliable and valid tool for assessing experiential avoidance. It has potential utility in research and clinical settings, such as monitoring patient progress in ACT-based interventions.

## Introduction

Avoidance is a common coping mechanism used to manage distress, encompassing two interrelated yet distinct concepts: experiential avoidance and behavioral avoidance [1]. While behavioral avoidance involves efforts to evade or alter external stressful situations that may trigger unpleasant internal states, experiential avoidance refers to the reluctance to remain in contact with private experiences—such as bodily sensations, emotions, thoughts, and memories—and the attempt to change the form or frequency of these experiences [2]. Thus, experiential avoidance consists of two main components: the avoidance of painful internal experiences (e.g., negative emotions, distressing thoughts, or intrusive memories) and the effort to avoid situations or contexts that may trigger these undesirable experiences [3, 4].

This distinction between the two types of avoidance is important because, while behavioral avoidance may provide short-term relief, experiential avoidance can lead to additional suffering [5] and has different long-term consequences [6]. Although avoidance has traditionally been central to the functional analysis of behavior and learning-based explanations of the emergence and maintenance of disorders [7], the emphasis on experiential avoidance has increased in recent years, especially with the paradigm shift in behavioral sciences promoting a transdiagnostic understanding of psychopathology [8].

Experiential avoidance is a core concept in the psychological flexibility model and acceptance and commitment therapy (ACT) [9]. It plays a critical role in the development, onset, and maintenance of various mental health issues and psychopathological disorders [8, 10, 11].

While avoidance may offer temporary relief, it often reinforces avoidance behaviors and exacerbates psychological distress over time, ultimately impairing daily functioning, personal goals, and engagement in life [12, 13].

The significance of experiential avoidance extends beyond individual well-being, as it can manifest in a variety of symptoms in the context of psychological disorders [14]. Akbari et al. [15] showed through a systematic review and meta-analysis that experiential avoidance is a transdiagnostic and transcultural factor contributing to the etiology of depression, anxiety, OCD-related, and post-traumatic stress disorders. It has also been strongly associated with nonsuicidal self-harm, suicidal experiences, emotional disorders, and online addictions [15–20]. These relationships may be explained by the direct effects of experiential avoidance, as well as its interactions with underlying mechanisms such as anxiety sensitivity, behavioral avoidance, and emotional regulation [21–23].

Furthermore, experiential avoidance is a central concept in the psychological flexibility model, which underpins ACT. This model emphasizes the importance of accepting and being present with internal experiences rather than avoiding them. As a core process in the development of psychological inflexibility, addressing experiential avoidance is crucial for treating various mental health conditions [2, 24]. Research has shown that reducing experiential avoidance is central to the effectiveness of various treatments, not only ACT but also mindfulness- and self-compassion-based interventions [25–27].

Given the increasing recognition of experiential avoidance’s role in mental health conditions and its central role in therapeutic models such as ACT, there is a growing need to assess and address experiential avoidance in both research and therapeutic contexts. However, as with many psychological constructs, experiential avoidance is inherently private, with few manifest indicators, necessitating a latent variable approach to its assessment [28]. Furthermore, the overlap between experiential avoidance and other variables—particularly those under the scope of psychological flexibility—can lead to inaccurate assessments [24]. Some researchers have argued that current self-report tools for measuring experiential avoidance may assess something other than the intended construct [23, 29, 30].

Borgogna et al. [31] summarized criticisms of the most widely used questionnaires of experiential avoidance, such as the Acceptance and Action Questionnaire series [32, 33], the Multidimensional and Brief Experiential Avoidance Questionnaires [34, 35], and the experiential avoidance subscale of the Multidimensional Psychological Flexibility Inventory [36]. Criticisms include questionable face and construct validity, inadequate construction processes, and issues with length and practical use.

To address these limitations, Borgogna et al. [31] developed the Experiential Avoidance Rating Scale (EARS), a concise six-item, unidimensional scale designed to assess experiential avoidance in a psychometrically sound manner. The EARS retains the original definition of experiential avoidance [2], incorporates items from previous tools, and has been validated through large independent samples (n1 = 3,628, n2 = 1,413). Its concurrent, convergent, divergent, factorial, and incremental validities make it a promising solution for more accurate and efficient assessment of experiential avoidance.

“Experiential avoidance plays a key role in mental health conditions and is central in therapeutic models such as ACT. Therefore, the present study aimed to develop a Persian version of the EARS and evaluate its psychometric properties. A reliable and culturally appropriate measure of experiential avoidance for Persian-speaking populations will contribute to the growing body of literature on psychological flexibility and inflexibility and advance the understanding and treatment of mental health in Iran.

Although Persian translations of experiential avoidance measures such as the AAQ-II, BEAQ, and MPFI-Ex exist, these instruments present certain challenges for research and clinical application in Iran. The AAQ-II has faced criticism regarding construct purity and susceptibility to measuring distress rather than avoidance per se [29], whereas the BEAQ’s multidimensional format and 15-item length may reduce its practicality for rapid assessment. The MPFI-Ex subscale, although theoretically comprehensive, embeds experiential avoidance within a larger framework of inflexibility, making it less efficient as a stand-alone screening tool. In contrast, the EARS offers a brief (6-item), unidimensional, and psychometrically robust alternative that aligns closely with the original conceptualization of experiential avoidance [2]. In Iran’s busy clinical environments—where time constraints, high caseloads, and limited mental health resources are common—such brevity and clarity can facilitate routine assessment, early identification of at-risk individuals, and progress monitoring in interventions such as ACT. Furthermore, developing a Persian EARS addresses potential cultural challenges in assessing experiential avoidance, such as differences in emotional expression norms influenced by Iranian cultural values (e.g., emphasis on stoicism or family-oriented coping), which may affect how avoidance is reported or interpreted compared with Western samples [37]. Critically, translating and validating a scale originally developed in Western contexts requires careful consideration of linguistic nuances and cultural relevance, including potential shifts in item meaning during translation that could impact construct validity. By providing a validated Persian EARS, this study not only fills a gap in culturally adapted tools but also enhances practical applications in Iranian mental health settings, such as integrating it into ACT-based therapies for disorders such as anxiety and depression, where experiential avoidance is a key transdiagnostic factor [15].

## Method

### Participants

This cross-sectional study evaluated the psychometric properties of the Persian-translated Experiential Avoidance Rating Scale (EARS). The sample consisted of 700 university students from Tehran, with a mean age of 27.71 years (SD = 9.35), 77.9% of whom were women. Participants were recruited from various universities in Tehran between April and June 2024.

### Procedure

The study received approval from the Student Research Committee at Kharazmi University (approval code: IR.KHU.REC.1403.119). Before participation, research assistants explained the study’s purpose and ensured participants that their responses would remain confidential. Informed consent was obtained, ensuring that the participants were fully aware of their rights and the study’s objectives. The participants completed a series of online questionnaires, including the EARS, AAQ-II, BEAQ, MPFI, PHQ-9, GAD-7, PCL-5, DUDIT, GHQ-12, BFI-N, PANAS-SF, MAAS, and MRNI-VR. To be included in the study, the participants had to be university students and willing to participate.

The Persian version of the EARS was developed via a forward–backward translation process. Initially, three bilingual experts fluent in both English and Persian independently translated the scale into Persian. Two psychologists then reviewed the translations for cultural relevance and clarity, resolving discrepancies through discussion. This review included addressing cultural nuances, such as ensuring that items reflected Persian idioms for emotional suppression (e.g., avoiding direct translations that might imply behavioral rather than internal avoidance), and considering societal norms around emotional expression in Iran. The translated version was subsequently back-translated into English by two additional bilingual translators who were unfamiliar with the original scale. The back-translated version was compared with the original version to ensure conceptual equivalence. A pretest with 71 participants was conducted to assess clarity and comprehension, leading to minor adjustments for readability. The pilot test results are further discussed in the Data Analysis section. To mitigate participant burden from the extensive battery of questionnaires (totaling 202 items), surveys were administered online with optional breaks, and attention-check items were included to monitor response quality.

### Measures

This study uses a total of 12 additional questionnaires, comprising 202 items. Of these, two complete scales and one subscale are specifically used to assess the concurrent validity of the EARS in relation to other experiential avoidance measures. Additionally, nine scales are employed to evaluate convergent validity with conceptually distinct yet related constructs, whereas one scale is used to assess discriminant validity with a distinct construct.

#### The Experiential Avoidance Rating Scale (EARS)

This six-item scale was developed by Borgogna et al. [31] on the basis of a conceptual definition of experiential avoidance. The authors aimed to address the conceptual and psychometric shortcomings of prior instruments. Each item on this scale inquires about individuals’ experiences over the past two weeks, with responses measured on a five-point Likert scale ranging from “never” (1) to “almost always” (5). The authors provided evidence for the scale’s conceptual, factorial, concurrent, convergent, and discriminant validity in their study [31]. The Persian version of this scale, utilized in the present research, was produced through a forward–backward translation process and was approved by one of the original authors (Nicolas Borgogna). The current study aims to assess the psychometric indicators of this scale.

#### The Acceptance and Action Questionnaire (AAQ-II)

The AAQ-II, developed by Bond et al. [32], is an improved version of the original questionnaire (AAQ) developed by Hayes et al. [33]. The AAQ-II consists of 10 items that measure acceptance, psychological flexibility, and experiential avoidance. Responses are provided on a seven-point Likert scale ranging from 1 (never) to 7 (always). Higher scores indicate greater psychological flexibility and acceptance, as well as lower levels of experiential avoidance. The results from 2,181 participants revealed that the AAQ-II has high construct validity and internal consistency. Cronbach’s alpha varied from 0.78 to 0.88 (M = 0.84), and test‒retest reliability over intervals of 3 and 12 months has also been reported (*r* = 0.81,* r* = 0.79) [32]. In Iran, Abbasi et al. [38] reported satisfactory internal consistency and split-half reliability for this model (ranging from 0.71–0.89 across four different samples). Their study also confirmed the two-factor model of this questionnaire and demonstrated its convergent validity through correlations with anxiety, depression, emotional regulation difficulties, and distress indicators and its discriminant validity between clinical and nonclinical groups [38]. In the present study, similar to the research of Borgogna et al. [31], this instrument will be used to examine concurrent validity.

#### Brief Experiential Avoidance Questionnaire (BEAQ)

This scale, developed by Gámez et al. [35], aims to condense and optimize the multidimensional experiential avoidance scale and consists of 15 items rated on a six-point Likert scale (from strongly disagree to strongly agree) [35]. Izadi-Mazidi [39] reported a Cronbach’s alpha of 0.66 and good fit with the intended one-factor structure for the Persian version of this instrument. In the current study, this tool is employed to assess concurrent validity [39].

#### The Multidimensional Psychological Flexibility Questionnaire (MPFI)

Developed by Rolffs et al. [36], this 60-item questionnaire assesses psychological flexibility and its six subdimensions (including acceptance, present-moment awareness, self-as-context, defusion, values, and committed action) as well as psychological inflexibility and its six subdimensions (including experiential avoidance, lack of present-moment awareness, self-as-content, cognitive fusion, lack of connection with values, and inaction). Each of these 12 subdimensions contains five items rated on a six-point Likert scale from 1 (strongly disagree) to 6 (strongly agree) [36]. The psychometric properties of the Persian version of this instrument were evaluated by Azadfar et al. [40], who confirmed its factor structure and internal consistency (Cronbach’s alpha of 0.86) and demonstrated its convergent validity through correlations with anxiety, depression, and self-compassion [40]. In the present study, the experiential avoidance subscale of this questionnaire will be utilized to examine concurrent validity, whereas other subscales, including the self-as-content subscale, will be used to assess convergent validity.

#### Patient Health Questionnaire (PHQ-9)

This nine-item instrument was designed by Löwe et al. [41] to assess symptoms of major depression (such as “feeling down, depressed, or hopeless”). Responses are based on a four-point Likert scale ranging from 0 (not at all) to 3 (nearly every day) [41]. To date, Kalibatseva and Lester (2018) reported a Cronbach’s alpha of 0.88, satisfactory test–retest reliability, and concurrent validity of the Persian version of this questionnaire with other depression assessment scales in outpatients [42]. In the present study, this instrument was used to assess the convergent validity of the questionnaire.

#### Generalized Anxiety Disorder 7-item scale (GAD-7)

Developed by Spitzer et al. [43], this seven-item scale is designed to assess symptoms of generalized anxiety disorder. The respondents indicated how often they had been bothered by symptoms such as “worrying excessively about different things” over the past two weeks. Higher scores indicate greater levels of anxiety [43]. Naeinian et al. [44] reported acceptable Cronbach’s alpha and test‒retest reliability (0.85 and 0.48) for the Persian version of this questionnaire and confirmed its one-factor structure and concurrent validity [44]. In the present study, this scale was utilized to evaluate symptoms of generalized anxiety disorder and to examine the convergent validity of the Experiential Avoidance Rating Scale.

#### Post-Traumatic Stress Disorder Checklist (PCL-5)

The 20-item PTSD Checklist for the DSM-5 is one of the most commonly used tools for assessing PTSD in civilian populations. The respondents indicate how much they have been bothered by symptoms such as “recurrent disturbing dreams related to the stressful experience” over the past month. Each item is rated on a five-point Likert scale from 1 (not at all) to 5 (extremely), with higher scores indicating more severe PTSD symptoms [45]. The psychometric properties of the Persian version of this scale were evaluated in the general population by Varmaghani, Fathi-Ashtiani, and Poursharifi (2018), who reported satisfactory internal consistency, convergent validity, and discriminant validity. The Cronbach’s alpha in this study was 0.92 [46]. In the present study, this questionnaire was used to assess convergent validity.

#### Drug Use Disorders Identification Test (DUDIT)

This 11-item questionnaire was designed by Berman et al. [47] to assess drug use disorders. Responses to items 1–9 are given on a five-point Likert scale from 0–4, and responses to items 10–11 are scored as 0, 2, or 4. Higher scores indicate greater problems related to drug use [47]. The psychometric properties of the Persian version of the DUDIT were evaluated by Habibi et al. [48], who reported Cronbach’s alpha and split-half reliability scores of 0.81 and 0.68, respectively [48]. In the present study, this scale is used for assessing convergent validity.

#### General Health Questionnaire (GHQ-12)

Developed by Goldberg and Williams [49] to assess nonpsychotic psychological problems, this 12-item questionnaire asks respondents to report their recent experiences with various issues. Responses are provided on a four-point Likert scale ranging from 0 (not at all) to 3 (much more than usual) [49]. The Persian version of this questionnaire was translated by Montazeri et al. [50], who reported satisfactory internal consistency (Cronbach’s alpha of 0.87) and convergent validity with the Quality of Life Questionnaire. Additionally, their study confirmed a two-factor structure for the questionnaire [50]. In the present study, this questionnaire was used to assess convergent validity.

#### Big Five Personality Inventory – Neuroticism Subscale (BFI-N)

The neuroticism subscale of the Big Five Personality Inventory [51] consists of 8 items, and responses are provided on a five-point Likert scale (from 1: strongly disagree to 5: strongly agree) [51]. Minaei and Jafari (2022) examined the validity and reliability of the Persian version of this inventory in a sample of university students and reported adequate internal consistency for all five factors (Cronbach’s alpha for the neuroticism subscale: 0.76). The five-factor model, as well as the convergent and discriminant validity of the questionnaire, was confirmed in their study.

#### Positive and Negative Affect Schedule–Short Form (PANAS-SF)

Developed by Watson et al. [52], this scale consists of 20 words representing various emotions (10 positive and 10 negative). Participants are asked to rate the intensity of each emotion at the time of completing the test on a five-point Likert scale from 1 (very slightly or not at all) to 5 (extremely) [52]. Sarfarazpour [53] reported Cronbach’s alphas of 0.81 and 0.80 for the positive and negative affect subscales, respectively, and confirmed the convergent validity of the test with constructs such as psychological well-being [53]. This questionnaire was used to assess convergent validity in the present study.

#### Mindful Attention Awareness Scale (MAAS)

Brown and Ryan developed this 15-item questionnaire in 2003 to assess mindful awareness in relation to the present moment. The items are rated on a scale from 1 (almost always) to 6 (almost never). Higher scores indicate greater mindfulness [54]. Ghorbani, Watson, and Bart (2009) reported a Cronbach’s alpha of 0.81 for this scale in a sample of 723 Iranian university students [55]. In the present study, this questionnaire was used to assess convergent validity.

#### Male Role Norms Inventory – Very Short Form (MRNI-VR)

McDermott et al. [56] designed this five-item scale to assess beliefs in traditional masculinity. Since traditional masculinity is based on values that do not support the experience or expression of emotions, it seems necessary to differentiate this concept from experiential avoidance [56]. As no Persian version of this inventory was available, the authors conducted the translation and back-translation process themselves (Cronbach’s alpha = 0.78). This tool is used to assess discriminant validity.

### Statistical analysis

Data preparation, cleaning, and assessment of concurrent, convergent, and divergent validity were conducted via SPSS version 27. Confirmatory factor analysis for a single-factor model was performed via LISREL 8.50. To address item performance, additional sensitivity analyses were conducted to evaluate the impact of individual items on scale reliability and factor structure.

## Results

### Pilot study

The pilot study (*N* = 71) evaluated the data collection process and questionnaire user-friendliness. On the basis of feedback, the demographic questions were moved to the end of the survey, and four attention-check questions were added to identify inattentive responses. The Persian EARS showed acceptable internal consistency (Cronbach’s α = 0.76). Key concurrent validity correlations included strong associations with the Acceptance and Action Questionnaire-II (AAQ-II, *r* = 0.60) and moderate associations with the Brief Experiential Avoidance Questionnaire (BEAQ, *r* = 0.31) but no significant correlation with the Multidimensional Psychological Flexibility Inventory’s Experiential Avoidance subscale (MPFI-Ex, *r* = 0.07, *p *> 0.05). Convergent validity was supported by significant correlations with 16 of 20 indicators, notably with the PTSD Checklist (PCL-5, *r* = 0.54) and Negative Affect Scale (PANAS-N,* r* = 0.47). Divergent validity was confirmed by a nonsignificant correlation with the Male Role Norms Inventory (MRNI-VR, *r* = 0.13, *p* > 0.05). The detailed results are presented in Tables 1and 2. Factor analysis was not conducted due to the small sample size.

**Table 1:** Concurrent validity indicators in the pilot study

Variable	1	2	3	4
EARS	-	-	-	-
AAQ-II	0.60	-	-	-
MPFI-Ex	0.07*	0.13*	-	-
BEAQ	0.31	0.56	0.19*	-
M	13.17	32.32	17.99	48.68
SD	4.32	11.42	5.92	13.58
α	0.76	0.90	0.92	0.88
N	71	71	71	71

*EARS* Experiential avoidance rating scale, *AAQ-II* Acceptance and action questionnaire-II, *MPFI-EX* Multidimensional psychological flexibility inventory-experiential avoidance subscale, *BEAQ* Brief experiential avoidance questionnaire

All the correlations are significant at *p* < 0.01, except those marked with *, which are nonsignificant (*p* > 0.05)

**Table 2:** Convergent and Divergent Validity Indicators in the Pilot Study

V	1	2	3	4	5	6	7	8	9	10	11
1	-	-	-	-	-	-	-	-	-	-	-
2	-.19*	-	-	-	-	-	-	-	-	-	-
3	-.38	.50	-	-	-	-	-	-	-	-	-
4	-.41	.56	.77	-	-	-	-	-	-	-	-
5	-.36	.61	.59	.70	-	-	-	-	-	-	-
6	-.25*	.43	.66	.68	.59	-	-	-	-	-	-
7	-.22*	.36	.57	.67	.58	.78	-	-	-	-	-
8	.31	-.24*	-.29*	-.23*	-.13*	-.23*	-.25*	-	-	-	-
9	.48	-.34	-.40	-.36	-.45	-.28*	-.32	.63	-	-	-
10	.43	-.22*	-.29*	-.39	-.63	-.30	-.38	.31	.73	-	-
11	.37	-.27*	-.46	-.53	-.48	-.57	-.61	.58	.61	.58	-
12	.37	-.25*	-.41	-.50	-.54	-.45	-.56	.51	.65	.72	.81
13	.40	-.17*	-.34	-.38	-.46	-.31	-.33	.36	.72	.70	.63
14	.43	-.28*	-.32	-.36	-.43	-.21*	-.27*	.31	.73	.66	.50
15	.54	-.18*	-.25*	-.26*	-.37	-.18*	-.19*	.33	.72	.64	.47
16	.23*	-.23*	-.15*	-.25*	-.14*	-.38	-.23*	.17*	.30*	.21*	.37
17	.35	-.25*	-.42	-.43	-.51	-.45	-.51	.30*	.64	.64	.56
18	.30*	-.18*	-.23*	-.31	-.45	-.16*	-.29*	.36	.52	.56	.41
19	-.08*	-.06*	.27*	.31	.32	.32	.44	-.33	-.44	-.46	-.49
20	.47	-.47	-.59	-.50	-.54	-.47	-.43	.38	.70	.53	.42
21	-.34	.34	.54	.39	.37	.46	.42	-.43	-.43	-.18*	-.40
22	.13*	-.04*	-.14*	-.08*	.03*	-.04*	-.02*	.40	.19*	-.09*	.31
M	13.17	19.35	23.2	21.97	18.01	23.8	23.11	14.07	16.03	16.62	12.1
SD	4.32	5.27	4.81	5.65	6.18	5.29	6.13	4.38	7.33	7.41	5.8
α	.76	.78	.91	.9	.92	.92	.95	.66	.94	.92	.93
N	71	71	71	71	71	71	71	71	71	71	71

V	12	13	14	15	16	17	18	19	20	21	22
1	-	-	-	-	-	-	-	-	-	-	-
2	-	-	-	-	-	-	-	-	-	-	-
3	-	-	-	-	-	-	-	-	-	-	-
4	-	-	-	-	-	-	-	-	-	-	-
5	-	-	-	-	-	-	-	-	-	-	-
6	-	-	-	-	-	-	-	-	-	-	-
7	-	-	-	-	-	-	-	-	-	-	-
8	-	-	-	-	-	-	-	-	-	-	-
9	-	-	-	-	-	-	-	-	-	-	-
10	-	-	-	-	-	-	-	-	-	-	-
11	-	-	-	-	-	-	-	-	-	-	-
12	-	-	-	-	-	-	-	-	-	-	-
13	.71	-	-	-	-	-	-	-	-	-	-
14	.59	.80	-	-	-	-	-	-	-	-	-
15	.57	.80	.85	-	-	-	-	-	-	-	-
16	.24*	.31	.33	.33	-	-	-	-	-	-	-
17	.69	.75	.78	.70	.32	-	-	-	-	-	-
18	.51	.52	.47	.44	-.09*	.47	-	-	-	-	-
19	-.62	-.53	-.40	-.29*	.07*	-.61	-.48	-	-	-	-
20	.41	.62	.67	.64	.40	.69	.45	-.20*	-	-	-
21	-.35	-.37	-.36	-.34	-.33	-.43	-.30*	.14*	-.68	-	-
22	.04*	-.06*	-.01*	-.03*	.09*	-.12*	.03*	-.01*	-.03*	-.07*	-
M	15.34	8.72	5.77	45.24	1.25	17.28	23.56	33.75	23.73	58.79	7.75
SD	7.32	7.17	5.2	20.07	3.87	6.72	3.62	8.06	10.48	13.53	6.25
α	.95	.91	.92	.97	.88	.88	.35	.91	.95	.91	.78
N	71	71	71	71	71	71	71	71	71	71	71

1 = Experiential Avoidance Rating Scale (EARS);

2 = Acceptance subscale of Multidimensional Psychological Flexibility Inventory (MPFI-Ac);

3 = Present-Moment Awareness Scale (MPFI-PM);

4 = Self-as-Context Scale (MPFI-SCX);

5 = Defusion Scale (MPFI-D);

6 = Values Scale (MPFI-V);

7 = Committed Action Scale (MPFI-CA);

8 = Lack of Present-Moment Awareness (MPFI-LPM);

9 = Self-as-Content Scale (MPFI-SCT);

10 = Cognitive Fusion Scale (MPFI-Fu);

11 = Lack of Values (MPFI-VL);

12 = Inactivity (MPFI-I);

13 = Patient Health Questionnaire (PHQ-9);

14 = Generalized Anxiety Disorder Scale (GAD-7);

15 = Post-Traumatic Stress Disorder Checklist, DSM-5 (PCL-5);

16 = Drug Use Disorders Identification Test (DUDIT);

17 = General Health Questionnaire (GHQ-12);

18 = Big Five Inventory—Neuroticism Scale (BFI-N);

19 = Positive Affect (PANAS-P);

20 = Negative Affect (PANAS-N);

21 = Mindful Attention Awareness Scale (MAAS);

22 = Very Brief Male Role Norms Inventory (MRNI-VR)

All correlations are significant at p < 0.01, except those marked with *, which are nonsignificant (p > 0.05)

The EARS showed the strongest correlations with the Acceptance and Action Questionnaire-II (AAQ-II, *r* = 0.60) and the Brief Experiential Avoidance Questionnaire (BEAQ, *r* = 0.31). However, correlations with the experiential avoidance subscale of the Multidimensional Psychological Flexibility Inventory (MPFI-Ex) were not significant (*r* = 0.07, *p* > 0.05). In terms of convergent validity, 16 out of 20 validity indicators showed significant correlations, with the strongest associations found with the Post-Traumatic Stress Disorder Checklist (PCL-5, *r *= 0.54), the Self-as-Content subscale of the MPFI (MPFI-SCT, *r* = 0.48), and the Negative Affect Scale (PANAS-N, *r* = 0.47). Furthermore, the lack of significant correlation with the Very Brief Male Role Norms Inventory (MRNI-VR) supports the scale’s divergent validity. Owing to the limited sample size, factor analysis was not conducted during this phase of the study; however, this small sample size may have reduced the statistical power for detecting weaker correlations, such as with the MPFI-Ex, and limited the robustness of the preliminary psychometric evaluations.

### Main study

The main study (*N* = 700, mean age = 27.71, SD = 9.35, 77.9% female) excluded cases with more than two incorrect responses to validity-check questions. To explore potential sex differences, an independent t test was conducted. No significant gender differences were found in the EARS scores (t(694) = 0.22, *p* = 0.83). Demographic information for this sample is provided in Table 3.

**Table 3:** Demographic information

Variable	
Age	M = 27.71, SD = 9.35
Gender	
Female	*N* = 545, 77.9%
Male	*N* = 151, 21.6%
Other	*N* = 40,6%
Native Language	
Persian	*N* = 519, 74.1%
Other	*N* = 181, 25.9%
Marital Status	
Single	*N* = 409, 58.4%
Committed Relationship	*N* = 135, 19.3%
Married	*N* = 136, 19.4%
Divorced	*N* = 19, 2.7%
Widowed	*N* = 1, 0.1%
Education	
Bachelor’s Degree	*N* = 425, 60.7%
Master’s Degree	*N* = 193, 27.6%
Ph.D	*N* = 45, 6.4%
Professional Doctorate	*N* = 37, 5.3%
Employment Status	
Employed	*N* = 251, 35.9%
Unemployed	*N* = 449, 64.1%

### Factor structure

Confirmatory factor analysis (CFA) supported a single-factor model for the Persian EARS, which was consistent with the original scale [31]. The fit indices are presented in Table 4. Item 1 (“I have tried my best to avoid thinking about thoughts I dislike”) had a lower factor loading (β = 0.26) than the other items did (β = 0.68–0.79). Sensitivity analysis excluding item 1 slightly improved reliability (α = 0.83 from 0.81) while maintaining good model fit. Item 1 was retained to preserve consistency with the original EARS, with cultural implications discussed in the Discussion section. All factor loadings were significant at *P* < 0.01 (see Table 5).

**Table 4:** Fit indices for confirmatory factor analysis of the persian EARS

Model	χ^2^ (df)	CFI	TLI	RMSEA	SRMR
Single-factor model	41.81 (9)	0.98	0.96	0.072	0.027
Excluding Item 1	24.12 (5)	0.97	0.95	0.074	0.029

*CFI* Comparative fit index, *TLI* Tucker‒Lewis index, *RMSEA* Root mean square error of approximation, *SRMR* Standardized root mean square residual

All factor loadings are significant at *p* < 0.01

**Table 5:** Factor Loadings in the Main Study

Item	Β	SE (B)	B
1. (I have tried my best to get rid of the thoughts that I do not like)	0.26	1.07	1
2. (When unpleasant memories come to me, to eliminate them from my mind, I do things that I regret.)	0.70	0.58	2.63
3. (To be less sad, I have done things that I regret.)	0.79	0.46	3.12
4. (To control my feelings, I have done things that I am not proud of.)	0.68	0.67	2.70
5. (My feelings were so painful that to get rid of them I even put in danger things that were important to me.)	0.71	0.71	3.03
6. (If there was a thing that I did not want to think about, I tried many ways to eliminate it from my mind, even if those ways were destructive.)	0.77	0.56	3.22

All factor loadings are significant at *P* < 0.01

### Concurrent, convergent, and divergent validity

The Persian EARS demonstrated good internal consistency (α = 0.81). The key concurrent validity findings included strong correlations with the AAQ-II (*r* = 0.60) and BEAQ (*r* = 0.40), with a weaker but significant correlation with the MPFI-Ex (*r* = 0.14, *p* < 0.05). Convergent validity was supported by strong associations with PTSD symptoms (PCL-5, *r* = 0.55), self-as-content (MPFI-SCT, *r *= 0.51), and negative affect (PANAS-N, *r* = 0.49). Divergent validity was confirmed by a nonsignificant correlation with traditional masculinity norms (MRNI-VR, *r* = 0.09, *p* > 0.05). The full correlation details are provided in Tables 6 and 7.

**Table 6:** Concurrent Validity Correlations (Main Study)

Variable	EARS	AAQ-II	MPFI-Ex	BEAQ
EARS	-	0.60**	0.14*	0.40**
M	14.66	32.45	18.02	48.72
SD	4.85	11.38	5.89	13.61
α	0.81	0.90	0.79	0.88
N	700	700	700	700

*EARS* Experiential avoidance rating scale, *AAQ-II* Acceptance and action questionnaire II, *MPFI-Ex* Multidimensional psychological flexibility inventory experiential avoidance subscale, *BEAQ* Brief experiential avoidance questionnaire

^*^p < 0.05

^**^p < 0.01

**Table 7:** Convergent and Divergent Validity Correlations (Main Study)

Variable	EARS	PCL-5	MPFI-SCT	PANAS-N	MRNI-VR
EARS	-	0.55**	0.51**	0.49**	0.09
M	14.66	45.30	16.05	23.75	7.80
SD	4.85	20.10	7.35	10.50	6.28
α	0.81	0.97	0.94	0.95	0.78
N	700	700	700	700	700

*EARS* Experiential avoidance rating scale, *PCL-5* PTSD checklist for DSM-5, *MPFI-SCT* Multidimensional psychological flexibility inventory self-as-content subscale, *PANAS-N* Positive and negative affect schedule negative affect subscale, *MRNI-VR* Male role norms inventory very short form

^**^p < 0.01

This study assessed the concurrent, convergent, and divergent validity of the Persian version of the Experiential Avoidance Rating Scale (EARS), which uses indices similar to those in Borgogna et al. [31]. The Cronbach’s alpha for the 6-item EARS was acceptable (α = 0.81) in this phase. Table 4 presents indicators of concurrent validity, while Table 5 displays those for convergent and divergent validity. With a sample size of 700, correlations above *r* = 0.10 were significant at *p* < 0.05, and those above *r* = 0.13 were significant at *p* < 0.01, unless otherwise noted.

As shown in Table 4, the EARS exhibited the highest correlations with the Acceptance and Action Questionnaire-II (AAQ-II, *r* = 0.60) and the Brief Experiential Avoidance Questionnaire (BEAQ, *r* = 0.40), supporting its concurrent validity. The correlation with the MPFI experiential avoidance subscale (MPFI-Ex) was weaker but significant (*r* = 0.14, *p* < 0.05). Convergent validity indicators, shown in Table 6, were all significant in the expected directions, with the strongest correlations found with the Post-Traumatic Stress Disorder Checklist (PCL-5, *r* = 0.55), the Self-as-Content subscale of the Multidimensional Psychological Flexibility Inventory (MPFI-SCT, *r* = 0.51), and the Negative Affect subscale (PANAS-N, *r* = 0.49). Furthermore, the lack of a significant correlation with the Very Brief Male Role Norms Inventory (MRNI-VR, *r* = 0.09, *p* > 0.05) supports the divergent validity of the EARS. While divergent validity was assessed via only one measure, this choice was based on its conceptual distinctness from experiential avoidance; future studies could incorporate additional unrelated constructs to broaden this evidence.

## Discussion

This study provides strong evidence for the psychometric validity and reliability of the Persian version of the Experiential Avoidance Rating Scale (EARS), establishing it as a valuable tool for assessing experiential avoidance in Persian-speaking populations. The findings align with research on the original EARS, confirming experiential avoidance as a unidimensional construct that can be reliably measured across cultural contexts [31, 35]. This enhances the scale's generalizability and underscores the robustness of experiential avoidance as a psychological construct.

### Psychometric robustness and reliability

The Persian EARS showed high internal consistency (Cronbach’s α = 0.81). Confirmatory factor analysis also supported a single-factor structure, indicating reliable assessment among Iranian university students. However, item 1 ("I have tried my best to avoid thinking about thoughts I dislike") had a low factor loading (β = 0.26), potentially because of cultural nuances where the suppression of unwanted thoughts is viewed as positive self-control in Iranian contexts [37]. Linguistic factors, such as the translation's verb choice ("get rid of"), may also shift its meaning toward overt behaviors rather than internal avoidance. Sensitivity analysis excluding item 1 improved reliability (α = 0.83) and maintained good model fit (χ^2^(5) = 24.12, CFI = 0.97, RMSEA = 0.074). Despite this, the item was retained to align with the original scale. Future research should explore rephrasing, such as "push away thoughts I dislike," to better capture the construct while preserving unidimensionality.

### Validity evidence

#### Concurrent validity

The Persian EARS demonstrated concurrent validity through significant correlations with the Acceptance and Action Questionnaire-II (AAQ-II, *r* = 0.60) and the Brief Experiential Avoidance Questionnaire (BEAQ, *r* = 0.40), indicating alignment with established measures of psychological inflexibility [32]. However, the weaker correlation with the Multidimensional Psychological Flexibility Inventory Experiential Avoidance subscale (MPFI-Ex, *r* = 0.14, *p* < 0.05) may reflect differences in construct focus: the MPFI-Ex assesses broader inflexibility, including cognitive fusion and value disengagement, whereas the EARS targets specific avoidance behaviors [36]. Methodological factors, such as varying Likert scales (5-point vs. 6-point) and MPFI-Ex's lower reliability in this sample (α = 0.79), could also attenuate the association. This suggests that EARS captures unique facets of experiential avoidance, which is consistent with prior modest associations in multidimensional measures [31].

#### Convergent validity

Convergent validity was supported by correlations with the PTSD Checklist (PCL-5,* r* = 0.55), the Negative Affect subscale of the PANAS (*r* = 0.49), and the MPFI self-as-content subscale (*r* = 0.51), which link experiential avoidance to emotional distress, trauma responses, and self-as-content processes [57–59].

#### Divergent validity

The nonsignificant correlation with the Very Brief Male Role Norms Inventory (MRNI-VR, *r* = 0.09, *p* > 0.05) confirms divergent validity, distinguishing experiential avoidance from traditional gender roles, despite potential overlaps in emotional suppression [57, 60].

### Theoretical and practical implications

Theoretically, these results support the universality of experiential avoidance and its role in psychological distress, aligning with acceptance and commitment therapy (ACT) principles [57, 61]. Practically, the Persian EARS enables the identification of at-risk individuals in clinical and research settings, facilitates early interventions, tracks changes in ACT-based therapies, and promotes evidence-based practices in Persian-speaking communities [58, 61].

### Limitations and future directions

Limitations include the sample's composition (primarily young, urban, female university students from Tehran; mean age = 27.71, 77.9% female), which may limit generalizability to males, older adults, rural populations, or diverse socioeconomic groups, where cultural norms around emotional expression differ. No gender differences emerged in the EARS scores (t(694) = 0.22, *p* = 0.83), but the imbalance warrants caution. The cross-sectional design prevents causal inferences, self-reports measure risk biases such as social desirability or common method variance [62], and the lack of test–retest reliability hinders assessments of temporal stability. The small sample size of the pilot study precluded factor analysis.

Future directions include replicating in diverse samples (e.g., clinical groups, older adults, Persian speakers outside Iran), incorporating longitudinal designs, test‒retest evaluations, and factor analyses with larger samples. Exploring the impact of experiential avoidance on positive outcomes such as resilience and well-being [63] and integrating the EARS into acceptance-based therapy studies could further elucidate its role in adaptive functioning [61].

## Conclusion

In conclusion, this study validates the Persian EARS as a reliable and valid tool for assessing experiential avoidance, addressing the need for culturally adapted instruments [35]. This study advances the understanding of the cross-cultural manifestations of experiential avoidance and supports its integration into research and clinical practice, fostering culturally inclusive mental health interventions in Persian-speaking populations.

## Data Availability

No datasets were generated or analysed during the current study.
